# STar-DETR: A Lightweight Real-Time Detection Transformer for Space Targets in Optical Sensor Systems

**DOI:** 10.3390/s25041146

**Published:** 2025-02-13

**Authors:** Yao Xiao, Yang Guo, Qinghao Pang, Xu Yang, Zhengxu Zhao, Xianlong Yin

**Affiliations:** 1 Shandong Key Laboratory of Space Debris Monitoring and Low-Orbit Satellite Networking, Qingdao University of Technology, Qingdao 266520, China; xyxyxy0814@126.com (Y.X.); p490789243@163.com (Q.P.); zhaozhengxu@qut.edu.cn (Z.Z.); yinxianlong0805@163.com (X.Y.); 2 National Astronomical Observatories, Chinese Academy of Sciences, Beijing 100101, China; yangxu@nao.cas.cn

**Keywords:** space targets, object detection, RT-DETR, lightweight, data augmentation

## Abstract

Optical sensor systems are essential for space target detection. However, previous studies have prioritized detection accuracy over model efficiency, limiting their deployment on resource-constrained sensors. To address this issue, we propose the lightweight space target real-time detection transformer (STar-DETR), which achieves a balance between model efficiency and detection accuracy. First, the improved MobileNetv4 (IMNv4) backbone network is developed to significantly reduce the model’s parameters and computational complexity. Second, group shuffle convolution (GSConv) is incorporated into the efficient hybrid encoder, which reduces convolution parameters while facilitating information exchange between channels. Subsequently, the dynamic depthwise shuffle transformer (DDST) feature fusion module is introduced to emphasize the trajectory formed by space target exposure. Finally, the minimum points distance scylla intersection over union (MPDSIoU) loss function is developed to enhance regression accuracy and expedite model convergence. A space target dataset is constructed, integrating offline and online data augmentation techniques to improve robustness under diverse sensing conditions. The proposed STar-DETR model achieves an $AP_{0.5:0.95}$ of 89.9%, successfully detecting dim and discontinuous streak space targets. Its parameter count and computational complexity are reduced by 64.8% and 41.8%, respectively, highlighting its lightweight design and providing a valuable reference for space target detection in resource-constrained optical sensors.

## 1. Introduction

**Figure 1:**
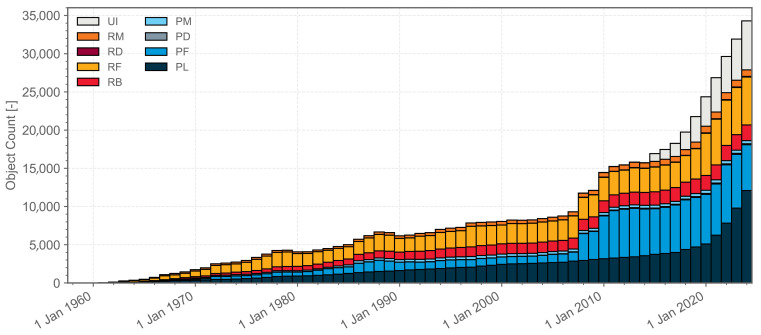
Evolution of a number of objects in geocentric orbit by object class, where PL means payload, PF means payload fragmentation debris, PD means payload debris, PM means payload mission related object, RB means rocket body, RF means rocket fragmentation debris, RD means rocket debris, RM means rocket mission related object, and UI means unidentified.

The exploration of space by humans has evolved over a period exceeding half a century. As the cost of space missions declines and space technology becomes increasingly sophisticated, countries worldwide are launching an increasing number of satellites into space. Figure 1 illustrates the evolution of a number of objects in geocentric orbit over the past few decades, categorized by object class [1]. According to the European Space Agency (ESA) [2], as of 20 September 2024, there are approximately 10,200 operational satellites in space. The Space Surveillance Network regularly tracks and records around 36,860 objects in space, and the total mass of all objects in Earth’s orbit has exceeded 13,000 tons. The rapid increase in the number of objects in space orbit has led to a significant rise in the risk of collisions between satellites and space debris or other non-cooperative satellites. The space environment has become increasingly hazardous, posing substantial risks and obstacles to space activities. In this paper, the term ‘space targets’ is used to refer primarily to space debris and certain non-cooperative satellites. The real-time detection of space targets is essential to prevent satellite collisions, as it provides real-time data for subsequent target tracking, cataloguing, trajectory prediction, and collision warning. This is a crucial step and an important component in ensuring the safety of the space environment.

**Figure 2:**
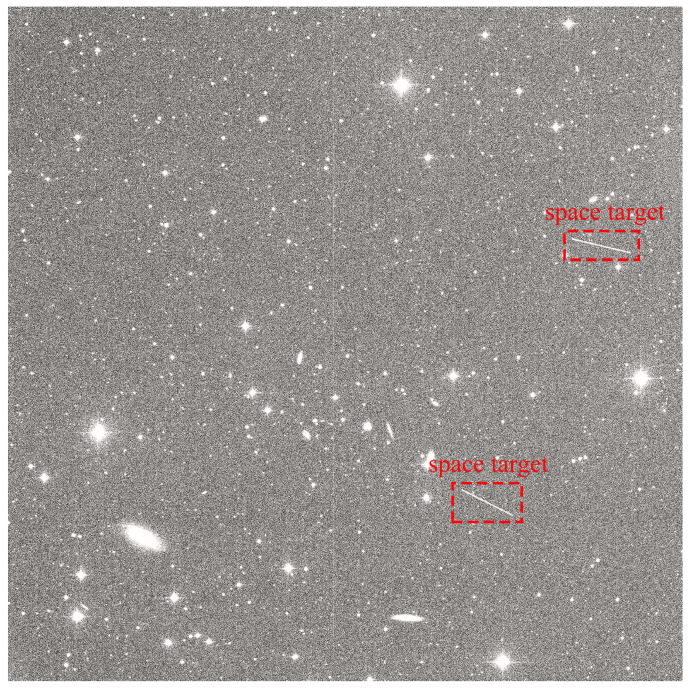
Streak imaging of moving space targets under exposure.

Space target monitoring systems can be classified according to the type of observation equipment, which includes optical sensors, radio telescopes, and radar systems [3]. Optical sensors offer a number of advantages, including intuitive observations, relatively high resolution (in the visible light spectrum), and a wider detection range. This enables them to cover larger areas of the sky and rapidly obtain optical imaging of targets. In accordance with the characteristics of the observation environment, monitoring systems are classified into two categories: ground-based and space-based. Compared to their space-based counterparts, ground-based wide-field optical sensors are not required to operate in the extreme conditions of space. Therefore, they are more cost-effective in terms of both construction and operation. Furthermore, they are easier to upgrade and maintain, offering greater flexibility. As a result, ground-based optical sensor systems are widely used in a variety of observation projects and are the preferred choice for space target detection tasks. In this study, the space targets are observed using ground-based optical sensors in long-exposure mode. By collecting light from distant objects over an extended period, the motion of fast-moving space targets, such as satellites or debris, is captured as streaks in the orange single-frame images, effectively distinguishing them from the starry background. The length and thickness of these streaks are determined by the target’s speed, trajectory, and duration of exposure. However, space targets have small reflective surfaces, and some are located at significant distances from Earth, which results in them occupying only a limited number of pixels in ground-based optical sensor images [4]. Consequently, they appear to be faint and challenging to discern. In addition, sensors are subject to various imaging characteristics and working conditions. In practical applications, atmospheric interference, light pollution and weather conditions often exert a complex and uneven influence on the background of the star field. Images may contain a multitude of stars and halos and exhibit a low signal-to-noise ratio (SNR) [5]. These challenges present significant obstacles to space target detection tasks, as they make it difficult for space target detection algorithms to balance accuracy and efficiency. Ensuring space environment safety requires a high-precision and high-efficiency method to accurately and rapidly determine the location of observed space targets. Figure 2 illustrates the streak imaging of moving space targets under exposure. 

In response to the aforementioned challenges, this paper proposes a lightweight space target detection model, designated as space target real-time detection transformer (STar-DETR). It effectively handles various types of streaks by leveraging downsampling in the backbone network and multi-scale feature extraction in the encoding layers without requiring additional preprocessing. This enables robust detection under different target speeds and imaging conditions. The model has been designed with the objective of reducing the number of parameters and computational complexity while simultaneously enhancing detection speed and maintaining high accuracy. It is important to note that, due to the nature of space target detection datasets in long-exposure scenarios, the scope of this study is strictly limited to single-frame detection tasks.

Innovative methodologies are integrated at various stages of the process, including the backbone network, the efficient hybrid encoder, and the loss function. The main contributions of this paper are as follows:

1. The improved MobileNetv4 (IMNv4) architecture is proposed as the backbone network. IMNv4 not only extracts high-quality feature representations but also significantly reduces the number of parameters and computational costs, thereby meeting the demand for lightweight design.
2. A novel lightweight convolutional technique, group shuffle convolution (GSConv), is introduced in the efficient hybrid encoder. GSConv necessitates a reduced number of parameters in comparison to standard convolutions while maintaining high continuity of semantic information propagation. This enhances the model’s capacity to express non-linear features and facilitates more effective information exchange between channels.
3. A feature fusion module, the dynamic depthwise shuffle transformer (DDST), is proposed for use with the space target dataset. The DDST module is employed after the Concat operation to merge feature maps of different sizes. It is capable of focusing attention on the trajectory formed by the exposure of space targets, which effectively addresses the overfitting issue.
4. The minimum points distance scylla intersection over union (MPDSIoU) localization loss function is proposed, which considers angle cost, distance cost, shape cost, and IoU cost in a comprehensive manner. Furthermore, the IoU cost is modified with the aim of improving the accuracy of bounding box regression and accelerating model convergence.
5. The space target dataset is established, and both offline and online data augmentation techniques are combined to improve the model’s performance and robustness under diverse sensing conditions.
6. Ultimately, through a series of experiments, the most suitable baseline model is selected, and the efficacy of the proposed improvements is validated. The experimental results illustrate that STar-DETR exhibits superior performance in terms of both accuracy and efficiency.

The remainder of the paper is structured as follows: Section 2 introduces related works in the field of space target detection. Section 3 presents the methodology, which outlines the framework and details of the proposed model. Section 4 describes the experimental design and result analysis, validating the effectiveness of the proposed model. Section 5 provides a conclusion.

## 2. Related Works

### 2.1. Traditional Algorithms for Space Target Detection

The majority of traditional space target detection algorithms rely on manually designed feature extraction, employing two principal approaches: detect-before-track (DBT) and track-before-detect (TBD). In the case of images exhibiting relatively high SNR, the DBT method is typically employed. This entails performing detection on individual frames, followed by the association of inter-frame information. Liu et al. [6] used a multi-star tracker system to perform target localization, converting angular observation data into space coordinates. Subsequently, the Gaussian MMSE differential correction algorithm was applied to fuse multi-frame image-space target position data, thereby facilitating the solution of the orbit equation. Jiang et al. [7,8] improved the star map by implementing median filtering, dual-structure morphological filtering, and guided filtering for denoising and background suppression. Subsequently, the Hough transform and Kalman filtering were employed for the detection and tracking of space targets, with successful results obtained even in the presence of complex backgrounds. Su et al. [9,10] proposed a multi-level joint detection and tracking model, improving local feature contrast methods to extract potential small space targets from optical image sequences. They utilized the differences in motion states to suppress star targets and also introduced a local spatiotemporal registration method, which offers superior target enhancement and clutter suppression effects. The DBT method processes a limited amount of image information per frame, which makes it a relatively simple process that is easy to implement and can be carried out in real-time. However, it requires high-quality single-frame images. The presence of noise and occlusion can result in false or missed detections, which have a negative impact on subsequent tracking performance.

For images with a very low SNR, space targets may appear indistinguishable from the background noise in a single frame, making direct detection impossible. In such cases, the TBD method is typically employed. In this approach, optical sensors provide data to the tracker, which directly associate possible targets across multiple frames. During the association process, the tracker assesses the authenticity of the targets. This method allows for the detection and tracking of space targets even when they are obscured by noise in individual frames. Li et al. [11] proposed a spatiotemporal pipeline multistage hypothesis testing (SPMHT) method, which combined spatiotemporal information and applied MHT for target discrimination. The effectiveness of the algorithm was demonstrated through simulations and experiments. Chen et al. [12] presented a concise method for space-based infrared target detection based on local spatial–temporal matching (LSM) and constructed the spatial–temporal joint model to enhance the target and suppress strong clutter. Liu et al. [13] introduced a dynamic programming sliding window method to detect faint space targets under long exposure conditions. Du et al. [14] combined dynamic programming (DP) and MHT methods for infrared target detection and tracking. Suthakar et al. [15] introduced annotated resident space object (RSO) images and examined median frame differencing, adjacent frame differencing, proximity filtering and tracking, and a streak detection method. While the TBD method tracks a large number of potential targets, resulting in higher accuracy, it is computationally more complex, slower, and may have difficulty meeting real-time requirements.

### 2.2. Deep Learning Methods for Space Target Detection

In the 2012 ImageNet Large-Scale Visual Recognition Challenge (ILSVRC), AlexNet [16] won the championship with an overwhelming margin, marking a pivotal advancement in deep learning for image classification. This triumph also signalled the beginning of rapid advancements in deep learning over the following decade. Convolutional neural networks (CNNs) based on deep learning have since been applied to a range of tasks, including space target detection [17,18,19,20]. Jia et al. [21] enhanced the Faster R-CNN algorithm for the detection and classification of astronomical objects, achieving encouraging outcomes, particularly for faint targets. Abay et al. [22] employed a feature fusion pyramid network (FPN) for space target detection, attaining an impressive 92% F1 score. De Vittori et al. [23] proposed a U-Net-based image segmentation network for real-time extraction of target trajectories from optical images. Guo et al. [24] introduced a channel and spatial attention network for rapid segmentation of space targets, effectively focusing on relevant features in both spatial and channel dimensions. Chen et al. [25] employed a hybrid approach, integrating long short-term memory (LSTM) models with CNNs to achieve the detection and tracking of space targets.

In the last two years, the you only look once (YOLO) series and detection transformer (DETR) object detection models have been rapidly developed. Some improved space target detection models based on YOLO and DETR have been proposed accordingly. Yuan et al. [26] improved the YOLOv5 model by integrating cross-layer context, adaptive weighting, and spatial information enhancement modules, thereby facilitating the effective detection of small targets in space. Nevertheless, the YOLOv5 model continues to be hindered by the computational burden imposed by the NMS post-processing. Wang et al. [27] enhanced the DETR model by incorporating diverse attention mechanisms into the backbone network and encoder, thereby facilitating the detection of spacecraft and space debris. Guo et al. [4] proposed an enhanced YOLOv8-based method for space target detection, addressing the intricate celestial background conditions. By improving the processes of feature fusion and feature reconstruction, they achieved the high-precision detection of space targets in image datasets captured by wide-field optical sensors. However, there is still scope for further enhancements in the efficiency of the aforementioned space target detection models.

Existing studies have focused predominantly on enhancing detection accuracy through the incorporation of complex modules. While these improvements contribute to better performance, they often result in increased model complexity, excessive parameter redundancy, and higher computational load. These factors can negatively affect the deployment of models on sensors, as they lead to slower inference speeds and increased resource consumption. A balance between accuracy and efficiency is crucial to ensure that models can achieve high performance while remaining suitable for practical deployment in real-time applications.

## 3. Methodology

The transformer is a deep learning architecture based on the attention mechanism, originally designed for natural language processing tasks. The Facebook AI team [28] adapted the transformer for use in object detection, introducing the detection transformer (DETR), which enables end-to-end object detection. The real-time detection transformer (RT-DETR) model [29] represents an advancement over the traditional DETR model. As the inaugural real-time, end-to-end object detector, it attains accelerated processing of multi-scale features while preserving accuracy through the decoupling of attention-based intra-scale feature interaction (AIFI) and CNN-based cross-scale feature fusion (CCFF), thereby enhancing processing speed. Furthermore, the model incorporates uncertainty-minimal query selection and eliminates the NMS post-processing step, thereby demonstrating superior performance in terms of both speed and accuracy when compared to other mainstream you only look once (YOLO) models of a similar size.

**Figure 3:**
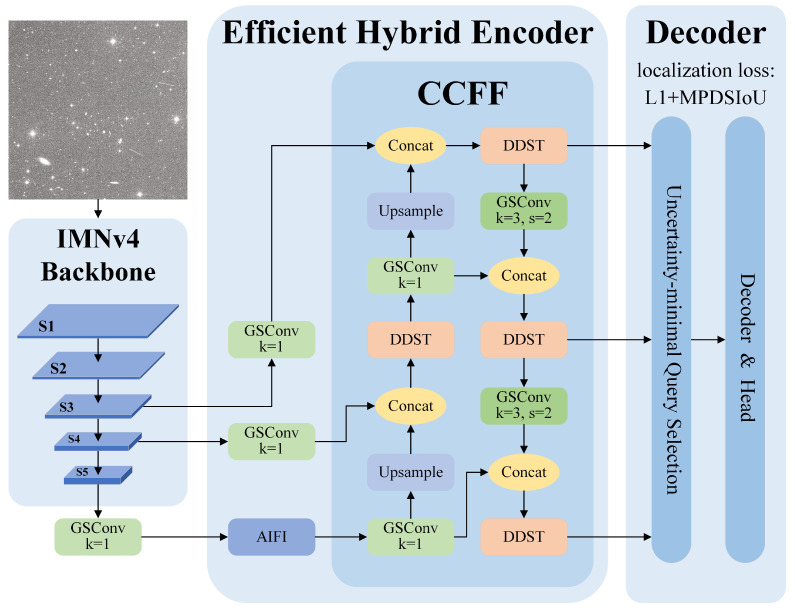
The structure of STar-DETR.

This paper proposes the STar-DETR model for space target detection based on RT-DETR. It preserves the excellent components of RT-DETR, including AIFI, CCFF, and the decoder, while implementing targeted enhancements to the backbone network, encoding, and the IoU loss function. The comprehensive structure is illustrated in Figure 3. The primary improvements of STar-DETR are as follows.

### 3.1. Improved MobileNetv4 Backbone

The RT-DETR model offers two distinct options for the backbone network: ResNet [30] and HGNetv2. The introduction of residual blocks to the ResNet architecture enables the direct transfer of input information to deeper layers of the network via skip connections. This addresses the issues of vanishing and exploding gradients that are inherent to deep learning models. HGNetv2 employs multiple HGBlocks and incorporates the uselab structure to improve upon the HGNet architecture. Both backbones have demonstrated considerable success in computer vision tasks. However, due to the utilization of a considerable number of full-channel standard convolutions (SC), the number of parameters and the computational load increase significantly in deeper layers, rendering it less suitable for integration into resource-constrained devices.

The Google team has put forth the MobileNet series of highly efficient models for mobile and embedded vision [31,32,33,34]. The models introduced techniques such as depthwise separable convolutions (DSConv), inverted residuals, and linear bottlenecks with the objective of reducing both the number of parameters and the computational load. This resulted in a higher memory efficiency while preventing information loss. In 2024, Google introduced MobileNetv4 [34], which proposed a further innovation in the form of a universal inverted bottleneck (UIB). In this paper, we present a lightweight improved MobileNetv4 (IMNv4) backbone for RT-DETR, inspired by MobileNetv4 and based on DSConv and UIB.

DSConv comprises two distinct operations: depthwise convolution (DW) and pointwise convolution (PW). The DW operation is employed for the extraction of spatial features, whereas the PW operation is utilized for the integration of channel features. To illustrate, the ratio of parameters and the ratio of computational load for a DSConv and a standard convolution (SC) are as follows:

(1) $$r_{Params}=\frac{Params_{\mathrm{DW}+\mathrm{PW}}}{Params_{\mathrm{SC}}}=\frac{K^{2}\times C_{in}+C_{in}\times C_{out}}{K^{2}\times C_{in}\times C_{out}}=\frac{1}{C_{out}}+\frac{1}{K^{2}},$$

(2) $$r_{FLOPs}=\frac{FLOPs_{\mathrm{DW}+\mathrm{PW}}}{FLOPs_{\mathrm{SC}}}=\frac{K^{2}\times H\times W\times C_{in}+H\times W\times C_{in}\times C_{out}}{K^{2}\times H\times W\times C_{in}\times C_{out}}=\frac{1}{C_{out}}+\frac{1}{K^{2}},$$

where *K* represents the convolution kernel size, *H* is the image height, *W* is the image width, $C_{in}$ is the number of input channels (also the number of DW kernels), and $C_{out}$ is the number of output channels (also the number of PW kernels). In deeper networks, $C_{out}$ is generally large. When using a 3 × 3 convolution kernel, the number of parameters and computational load associated with DSConv can be reduced to approximately 1/9 of that in an SC. This reduction is particularly notable as the network depth increases and the number of channels grows, resulting in a significant increase in the required number of convolution kernels. Consequently, DSConv can markedly reduce the number of parameters and the computational load in the feature extraction process, which is crucial for developing lightweight networks.

The UIB is composed of two optional DWs and two PWs. ${\mathrm{DW}}_{1}$ is situated prior to ${\mathrm{PW}}_{1}$, and ${\mathrm{DW}}_{2}$ is located between ${\mathrm{PW}}_{1}$ and ${\mathrm{PW}}_{2}$. By selecting different DW configurations, four distinct types of blocks can be obtained: ExtraDW, inverted bottleneck (IB), ConvNext, and feed-forward networks (FFN). Furthermore, a novel block, the Fused IB, has been devised by integrating a SC with a PW. The Fused IB is especially beneficial for downsampling and feature extraction in shallow networks.

**Figure 4:**
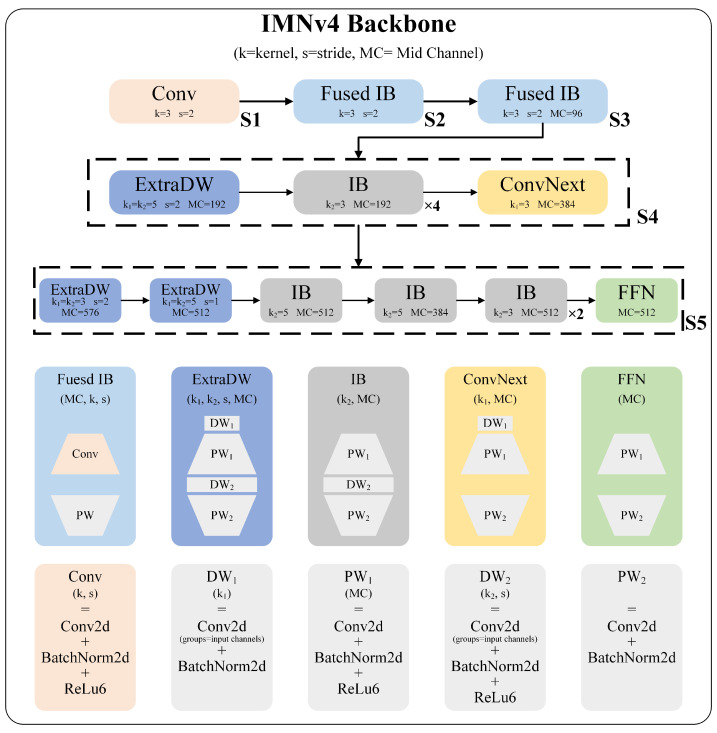
The structure of the IMNv4 backbone and the details of UIBs.

The backbone network of this paper is constituted of a flexible composition of different UIBs. The backbone network structure and the details of the UIBs are illustrated in Figure 4, wherein the trapezoids signify alterations in the number of channels. The stem layer utilizes an SC, whereas stage layer 1 and stage layer 2 employ fused IB for downsampling and feature extraction. Stage layer 3 and stage layer 4 are constituted by combinations of four distinct UIBs. Among these, the IB performs spatial mixing on expanded feature activations, thereby increasing the model capacity. The ConvNext approach employs DW prior to the expansion layer, facilitating spatial mixing with larger kernel sizes at a reduced computational cost. The ExtraDW synthesizes the benefits of both IB and ConvNext, employing a dual DW configuration to expand the receptive field. This design enhances the spatial mixing at the initial stage of the layer, effectively mitigating the issue of resolution loss. The final component is the FFN, which follows a sequence structure of PW + activation + PW. Its formula can be expressed as follows:

(3) $$FFN\left(x\right)=Act\left(xW_{1}+b_{1}\right)W_{2}+b_{2},$$

where $W_{1}$ and $W_{2}$ correspond to two successive PWs. By situating the FFN at the end of the backbone network, it is possible to integrate channel features and thereby enhance the feature extraction capability. Table 1, which corresponds to Figure 4, lists the details of the backbone structure and the number of parameters required for each layer.

### 3.2. Lightweight Group Shuffle Convolution

Following the extraction of features by the backbone network, the final three stages (S3, S4, and S5) are fed into the hybrid encoder. In the original RT-DETR model, the 1 × 1 SC is employed at the inputs of S3 and S4, as well as the intra-scale interaction of S5, with the objective of adjusting the number of channels and enhancing feature expressiveness. Furthermore, the 3 × 3 SC is utilized for downsampling in the CCFF during top-down feature fusion. Although SC typically requires more resources, convolution operations are indispensable during the encoding phase. Following an analysis and experimental validation, it was determined that replacing the 3 × 3 SC with DSConv in the hybrid encoding phase, with the objective of reducing parameters and computation, resulted in a notable decline in accuracy due to the prevalence of DSConv variants in the backbone.

**Figure 5:**
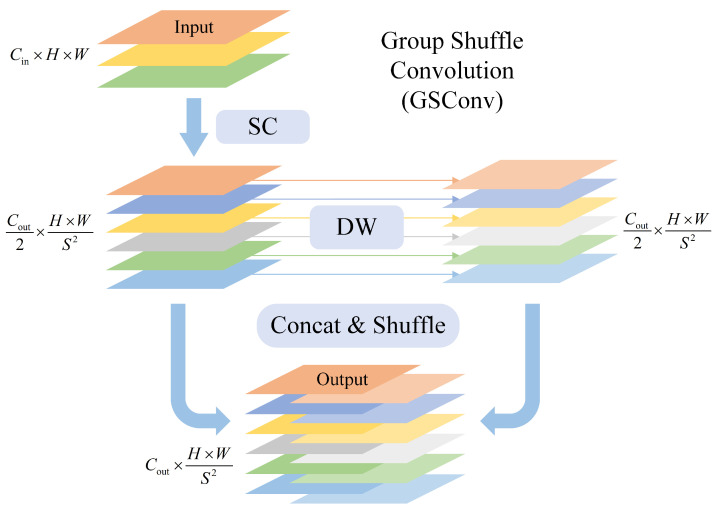
The structure of GSConv. “SC” denotes a standard convolutional 2D layer followed by batch normalization and activation. “DW” denotes a depthwise convolutional 2D layer followed by batch normalization and activation.

In order to address the trade-off between accuracy and lightweight design in hybrid encoding, the lightweight group shuffle convolution (GSConv) [35] is introduced, which considers the advantages of SC, DSConv, and channel shuffle [36,37] in a comprehensive manner. GSConv is a combination of SC and the DW from DSConv, as illustrated in Figure 5. The process commences with a single SC, wherein the output channel count is set to $C_{out}$/2. This is followed by DW, which serves to extract additional spatial features. Thereafter, the results of the two convolutions are concatenated, thereby restoring the output channel count to $C_{out}$. Ultimately, a channel shuffle operation is applied, ensuring that the corresponding channels from the two convolutions are placed together. The parameter and computation complexity of GSConv are as follows:

(4) $$Params_{\mathrm{GSConv}}∼O\left(K_{1}^{2}\times C_{in}\times \frac{C_{out}}{2}+K_{2}^{2}\times \frac{C_{out}}{2}\right),$$

(5) $$FLOPs_{\mathrm{GSConv}}∼O\left(\frac{H\times W}{S^{2}}\times Params_{\mathrm{GSConv}}\right),$$

where *S* represents the stride, and $K_{1}$ and $K_{2}$ are the kernel sizes for the SC and DW in GSConv, respectively. In this paper, $K_{1}$ is maintained at the same value as the kernel size of the original SC in the model, while $K_{2}$ is set to 5. A comparison of the parameter count and computational complexity of SC in Formulas (1) and (2) reveals that GSConv reduces both the parameter count and computational complexity by approximately 50%, effectively making the model more lightweight. Concurrently, GSConv maintains the high continuity of semantic information propagation intrinsic to standard dense convolutions, thereby enhancing the model’s capacity to express non-linear features and satisfying the accuracy requirements of the model.

### 3.3. Dynamic Depthwise Shuffle Transformer

In the context of object detection tasks, it is common to further integrate the concatenated features subsequent to the Concat operation. Among the most frequently utilized object detection algorithms in recent years, YOLOv5 employs a dual-branch C3 module subsequent to the Concat operation, which performs nonlinear transformations and spatial information integration. YOLOv8 introduces the C2f module, which incorporates identity mappings during feature mapping and utilizes a greater number of residual connections, thereby facilitating the more effective preservation of feature information and accelerating convergence. These methods primarily focus on improving feature expression with a view to enhancing accuracy, but there is still scope for further optimization in terms of model lightweighting. YOLOv9 [38] combines CSP [39] and ELAN [40] to design a lightweight network structure called GELAN. YOLOv10 builds upon C2f and introduces C2fCIB, where the conventional bottleneck is supplanted with a compact inverted bottleneck (CIB). The RT-DETR model deploys a reparameterized convolution module, RepC3, which enables the network to utilize diverse structures during training and inference. While YOLOv9, YOLOv10 [41], and RT-DETR all demonstrate an effective reduction in parameters and computational complexity, these works have been evaluated on general-purpose datasets. In light of the distinctive characteristics of space target datasets, there is scope for further optimization.

**Figure 6:**
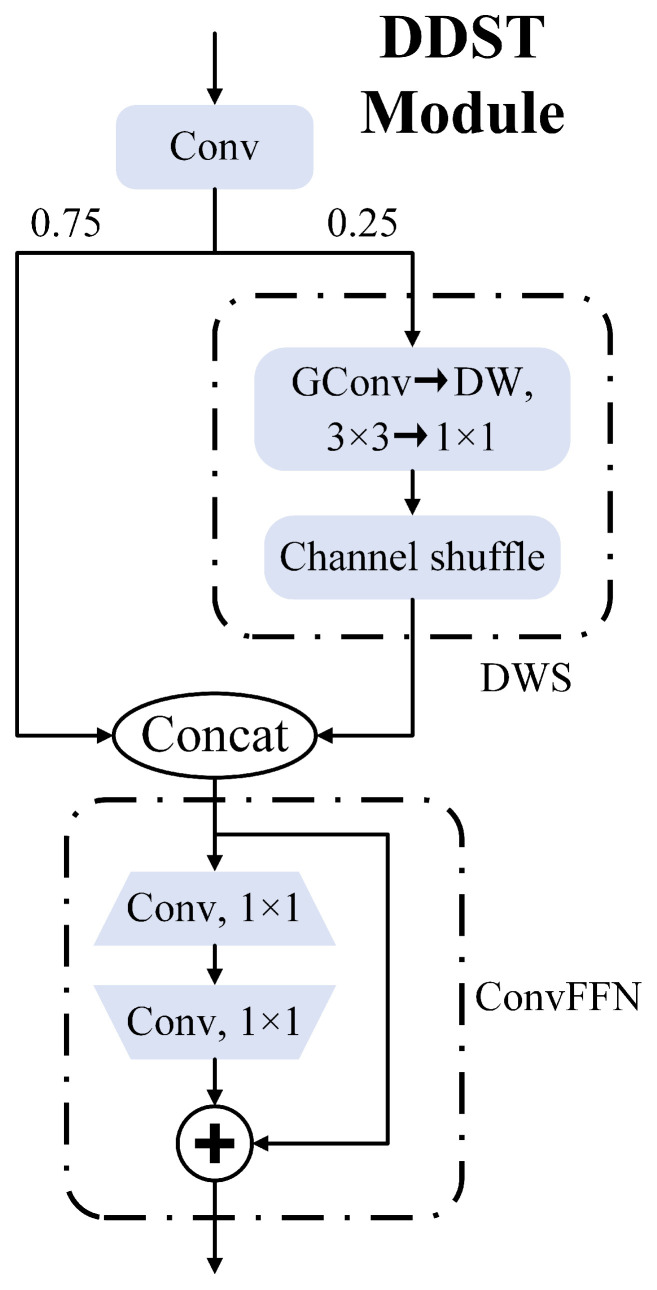
The structure of the DDST module.

A more suitable dynamic depthwise shuffle transformer (DDST) module for streak space target detection is proposed in this paper based on the dynamic group shuffle transformer (DGST) module [42]. The structural improvements are illustrated in Figure 6. The DDST module represents features in an efficient manner, integrating techniques such as group convolution [16], channel shuffle, FFN, and vision transformer while utilizing a dual-channel attention mechanism.

Let $X\in R^{C\times H\times W}$ denote the input feature map, where *C*, *H*, and *W* represent the number of channels, height, and width, respectively. The model initially performs a pointwise convolution, followed by a 3:1 splitting strategy, whereby only one-quarter of the channels undergo group convolution and channel shuffle operations. This process can be formulated as follows:

(6) $$X_{1},X_{2}=Split\left(Conv\left(X\right),ratio=3:1\right),$$

(7) $$X_{DWS}=Shuffle\left(DW_{1\times 1}\left(X_{2}\right)\right).$$

Finally, the two parts are concatenated and passed through a ConvFFN layer, producing the output feature map *Y*:

(8) $$Y=ConvFFN(Concat\left(X_{1},X_{DWS}\right)).$$

In this study, the objects to be detected are trajectories formed by the exposure of the space targets, which typically have low SNRs. This issue is particularly pronounced in the S5 detection layer following 32× downsampling, wherein the detected target occupies a mere fraction of the pixels. Theoretically, the DDST module offers the following advantages. First, the 3:1 split strategy and group convolution significantly reduce the number of model parameters and computational requirements while promoting inter-group information exchange and enhancing feature representation. Next, by adjusting the number of groups, the GConv is transformed into DW, and the DW kernel size is reduced to 1 × 1, thereby adapting to low SNR space targets and preventing overfitting. Finally, the ConvFFN layer, through a fully connected approach that first increases and then reduces dimensionality, allows the model to fully learn nonlinear features, which is particularly important for low SNR targets. The DDST module, an improved version based on the DGST module, performs exceptionally well on streak-like space targets, effectively focusing attention on the trajectory formed by space target exposure, resulting in more accurate target recognition.

### 3.4. Minimum Points Distance Scylla Intersection over Union

In the context of object detection, the loss function serves to quantify the discrepancy between the predicted information and the ground truth labels. This enables the direction of network training to be guided, thus facilitating the model’s ability to more accurately recognize and localize objects. Intersection over union (IoU) is a metric that represents the ratio of the intersection and union between the detection boxes and the ground truth (GT) boxes. It is frequently employed as the localization loss function in object detection models.

(9) $$IoU=\frac{\left|B\cap B^{gt}\right|}{\left|B\cup B^{gt}\right|},$$

where *B* and $B^{gt}$ represent the predicted detection box and the GT box, respectively. Various forms of IoU loss functions have been proposed, taking into account factors such as overlapping area, the distance between the key points of the boxes, and the width and height of the boxes. The RT-DETR baseline model employs both L1 loss and generalized-IoU (GIoU) loss [43] as its localization loss functions. In comparison to the conventional IoU definition presented in Equation (9), GIoU addresses the issue of zero gradients that arise when the bounding boxes do not intersect. Nevertheless, in the event that one box entirely encompasses the other, GIoU degenerates to standard IoU. Distance-IoU (DIoU) [44] considers the distance between the centers of the bounding boxes. Complete-IoU (CIoU) and efficient-IoU (EIoU) [45] further extend DIoU by incorporating the width and height information of the boxes. CIoU focuses on the aspect ratio of the boxes, while EIoU separates the width and height, computing the loss function based on the individual differences in width and height.

**Figure 7:**
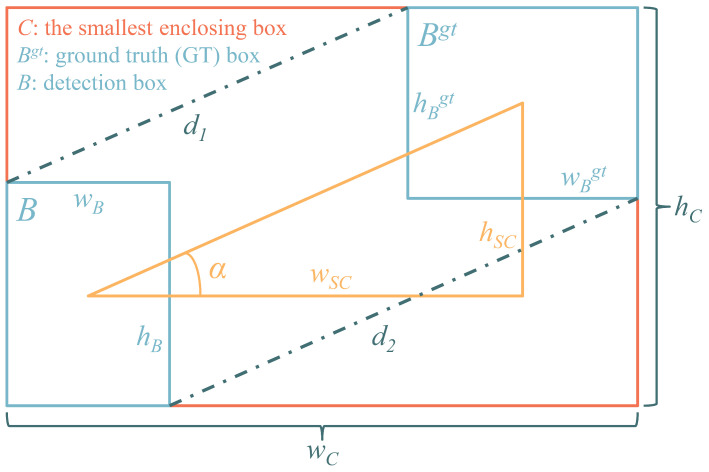
The visual interpretation of the meaning of the variables in the IoU loss function.

Scylla IoU (SIoU) [46] redefines the distance loss and shape loss and incorporates the angular relationship of the line connecting the centers of the detection box and the GT box. This approach introduces directional matching of the boxes, thereby reducing the degrees of freedom in the regression process and accelerating convergence. The SIoU loss function is defined as follows:

(10) $$L_{SIoU}=1-IoU+\frac{\Delta +\Omega }{2},$$

(11) $$\Lambda =\sin\left(2\alpha \right)=1-2\sin^{2}\left(\alpha -\frac{\pi }{4}\right),$$

(12) $$\Delta =\sum_{t=x,y}\left(1-e^{-\gamma \rho_{t}}\right),\gamma =2-\Lambda ,$$

(13) $$\left\{\begin{array}{c} \rho_{x}={\left(\frac{w_{SC}}{w_{C}}\right)}^{2}\\ \rho_{y}={\left(\frac{h_{SC}}{h_{C}}\right)}^{2} \end{array}\right.,$$

(14) $$\Omega =\sum_{t=w,h}{\left(1-e^{-\omega_{t}}\right)}^{\theta },\theta =4,$$

(15) $$\left\{\begin{array}{c} \omega_{w}=\frac{\left|w_{B}-w_{B}^{gt}\right|}{\max(w_{B},w_{B}^{gt})}\\ \omega_{h}=\frac{\left|h_{B}-h_{B}^{gt}\right|}{\max(h_{B},h_{B}^{gt})} \end{array}\right.,$$

where Λ represents the angle cost, Δ represents the distance cost, and Ω represents the shape cost. Figure 7 provides a visual representation of the interpretation of these variables within the formulas. As illustrated, SIoU employs a comprehensive approach, encompassing the angle cost, distance cost, shape cost, and IoU cost while maintaining the conventional IoU cost, as defined in Equation (9). The concept of the minimum points distance IoU (MPDIoU) [47] is introduced in this paper, which leverages the fact that two diagonal points of a rectangle in a plane can uniquely define the rectangle. Based on this, we propose the minimum points distance scylla IoU (MPDSIoU) loss function. By modifying the IoU cost term in Equation (10) and adjusting the weights of the various factors, we aim to further optimize the model’s convergence. The formula is as follows:

(16) $$L_{MPDSIoU}=1-MPDSIoU+\eta \left(\Delta +\Omega \right),$$

(17) $$MPDSIoU=\frac{\left|B\cap B^{gt}\right|}{\left|B\cup B^{gt}\right|}-\eta \frac{d_{1}^{2}+d_{2}^{2}}{2({w_{C}}^{2}+{h_{C}}^{2})},$$

where $d_{1}$ and $d_{2}$ represent the distances between key points of the detection box and the GT box, as illustrated in Figure 7. The MPDSIoU cost directly considers the distance between two top-left corners and two bottom-right corners. This effectively complements the distance cost and shape cost, which incorporate the angle term, and provides a more comprehensive measure of the error between the detection and GT boxes. In terms of weight parameter settings, we aim to achieve the following:

- The weights for Δ, Ω, and the rectangular key point distance factors $\frac{d_{1}^{2}+d_{2}^{2}}{2(w_{C}^{2}+h_{C}^{2})}$ should be equal (denoted as η in Equations (16) and (17)).
- The final form of $L_{MPDSIoU}$ should be as concise and aesthetically pleasing as possible, meaning the sum of the coefficients for $\frac{d_{1}^{2}+d_{2}^{2}}{w_{C}^{2}+h_{C}^{2}}$, Δ, and Ω should be 1.

To meet these objectives, we solved η and obtained η=2/5. The final form of $L_{MPDSIoU}$ is expressed as follows:

(18) $$L_{MPDSIoU}=1-\frac{\left|B\cap B^{gt}\right|}{\left|B\cup B^{gt}\right|}+\frac{d_{1}^{2}+d_{2}^{2}}{5({w_{C}}^{2}+{h_{C}}^{2})}+\frac{2(\Delta +\Omega )}{5}.$$

The efficacy of the MPDSIoU loss in improving the accuracy of bounding box regression and accelerating the model’s convergence speed has been demonstrated in experimental studies.

## 4. Experiments and Analysis

### 4.1. Experimental Preparations

#### 4.1.1. Datasets and Data Augmentation

The images in this dataset are sourced from the Weihai Astronomical Observatory Xuanji Sky Survey Telescope and the open access observational image data from the Observatorio Astronómico de La Sagra in Spain. The original images have a resolution of 2560 × 2560 pixels, stored in ‘jpg’ format, with each pixel corresponding to 1.468 arcseconds. In the background, stars appear as point-like sources, whereas galaxies and nebulae are represented as blotchy or more complex shapes. The space targets to be observed manifest as streak patterns under exposure, allowing them to be distinguished from other objects and noise. The streak length ranges from 200 to 1500 pixels, with a wide distribution. The streak width is only 4 to 10 pixels, presenting a challenge for accurate detection. We selected approximately 5200 images containing streak-like targets from a large volume of observational data, encompassing around 6000 space targets in total. These streak-like space targets were then annotated using LabelImg, an open-source graphical image annotation tool that supports the creation of bounding boxes and the assignment of labels to objects in images. Specifically, for each visible space target, a rectangular bounding box was manually drawn and adjusted to tightly enclose the target while minimizing the inclusion of background areas. LabelImg automatically converts the annotations into YOLO format (<object-class> <x_center> <y_center> <width> <height>) for storage. Finally, the dataset was randomly divided into training, validation, and test sets in a ratio of 7:2:1.

In the field of computer vision, data augmentation [48] represents a pivotal technique that generates novel training samples through the application of transformations and expansions to the original dataset. This increases the diversity of the data, enhances the model’s robustness and generalization ability, and reduces the risk of overfitting. In the context of space applications, as discussed in this paper, data augmentation can effectively address the issue of data scarcity, which is caused by the difficulty in obtaining high-quality space target image data. Furthermore, it enables the simulation of diverse meteorological and environmental conditions, thereby facilitating the model’s capacity to adapt to variations across different scenarios.

**Figure 8:**
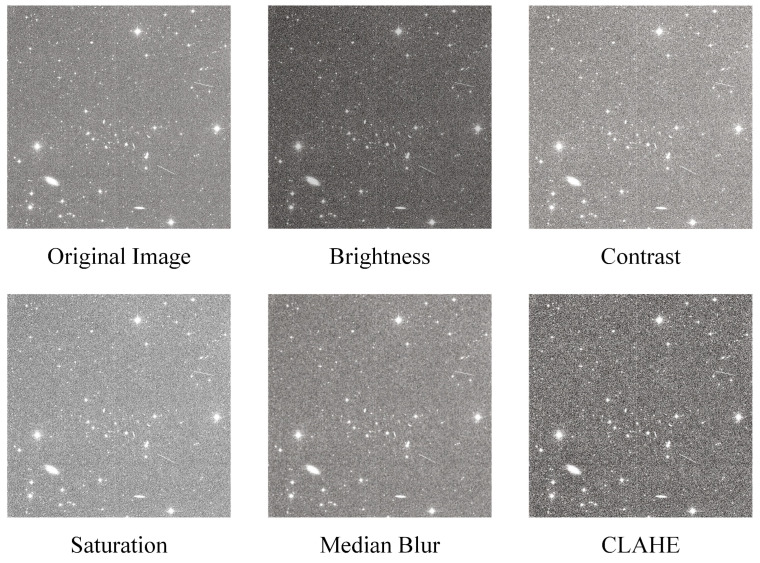
Random image adjustments in online data augmentation.

A methodology that integrates both offline and online data augmentation techniques is presented in this paper. Offline data augmentation is employed before training with the objective of augmenting the sample size. Each image in the dataset, along with its associated labels, is subjected to transformations such as rotation, flipping, stitching, and cropping (without compromising the integrity of the targets). These transformations expand the original dataset to approximately 32,000 images. During the training process, online data augmentation is employed to enhance sample diversity. The open-source Albumentations library [49] is employed, whereby in each training epoch, different images are randomly selected and adjusted, as illustrated in Figure 8. This encompasses the following:

- With a 2% probability, randomly adjust the brightness of the image;
- With a 2% probability, randomly adjust the contrast of the image;
- With a 2% probability, randomly adjust the saturation of the image;
- With a 2% probability, apply median blur to the image;
- With a 2% probability, apply contrast-limited adaptive histogram equalization (CLAHE) to the image.

The overall performance and stability of the model are enhanced through the combination of offline and online data augmentation.

#### 4.1.2. Experimental Settings

Table 2 illustrates the experimental environment and parameter settings for the RT-DETR-related experiments in this paper. All detection models in this study were trained under the same hardware and software environment, using identical training, validation, and test sets. During the experiments, the input image size was resized to 640 × 640 to facilitate feature extraction for space targets at various scales. In the training phase, AdamW [50] was employed as the optimizer for DETR-based models, while SGD was used for the remaining models. The initial learning rates of the compared models were consistent with those reported in their original papers, set to 0.001, 0.01, or 0.02, with a momentum of 0.9. Training for all models was terminated only when the accuracy converged.

#### 4.1.3. Evaluation Metrics

In this study, average precision (*AP*) is employed as the metric for evaluating the accuracy of the object detection model. *AP* is represented as the area under the *precision–recall* curve, and the calculation formula is as follows:

(19) $$AP=\int_{0}^{1}Precision\left(Recall\right)d\left(Recall\right),$$

where *precision* represents the probability that a sample predicted as positive is actually positive. *Recall* represents the probability that a positive sample is correctly predicted. The related calculation formulas are as follows:

(20) $$Precision=\frac{TP}{TP+FP},$$

(21) $$Recall=\frac{TP}{TP+FN}.$$

In single-class object detection, the symbols *T* and *F* are used to indicate whether a sample has been correctly predicted, while *P* and *N* represent whether the sample has been predicted as positive or negative. Specifically, *TP* denotes a correctly identified positive sample, *FP* represents a negative sample that has been erroneously classified as positive, and *FN* signifies a positive sample that has not been successfully detected. The criterion for determining the accuracy of detection is whether the IoU between the detection box and the GT box meets a specified threshold. In other words, the higher the IoU threshold, the more rigorous the criterion for determining whether a detection is correct. $AP_{0.5}$ represents the detection accuracy when the IoU threshold is set to 0.5 in the experiment. $AP_{0.5:0.95}$ represents the average of *AP* values calculated at IoU thresholds ranging from 0.5 to 0.95, with an interval of 0.05. The formula is as follows:

(22) $$AP_{0.5:0.95}=\frac{1}{10}\sum_{i=0}^{9}AP_{0.05i+0.5}.$$

In general object detection tasks, the primary metric for evaluating accuracy is often the $AP_{0.5}$. However, in the case of space target detection, each pixel in the image corresponds to tens or even hundreds of kilometers in space, necessitating the use of more rigorous evaluation metrics to ensure the accurate localization of space targets. In this paper, the more rigorous $AP_{0.5:0.95}$ metric is employed for the evaluation of model accuracy.

The model size is quantified in terms of the number of parameters (Params) and the size of the weight file. The inference computational cost is evaluated based on the number of floating point operations (FLOPs), while the inference speed is represented by frames per second (FPS) and inference time. Params and FLOPs metrics primarily assess the model’s suitability for deployment on resource-constrained devices, whereas FPS and inference time reflect the performance of the model in real-time application scenarios. The combination of these metrics allows for an evaluation of the model’s lightweight effect.

### 4.2. Baseline Model Experiments

In order to select the most suitable baseline model and investigate the effects of two hyperparameters, depth and width, on model size and accuracy, experiments were conducted using the space target dataset with RT-DETR models of varying scales. Based on the hyperparameter settings of the popular YOLO series models, further adjustments were made to the depth and width values in RT-DETR in order to develop new variants of the RT-DETR baseline model. The results of the experiment are presented in Table 3.

The imaging of a space target is distributed along the diagonal of the detection box, and the SNR within the box is low. Consequently, an excessive number of parameters in the model may result in the noise within the box being mistaken for features, leading to overfitting. Accordingly, in the experiment, despite the use of a greater number of parameters and computational resources by RT-DETR-L and RT-DETR-r34, their accuracy is slightly inferior to that of RT-DETR-r18. Based on RT-DETR-r18, the depth was adjusted to 0.33 and the width to 0.25, inspired by the hyperparameter tuning approach of YOLOv10n. This resulted in a 23.1% reduction in Params and a 40.2% reduction in FLOPs, while $AP_{0.5:0.95}$ only decreased by 0.8%, still meeting the requirements for space target detection. As a result, RT-DETR-r18 (depth = 0.33, width = 0.25) is selected as the baseline model in this paper.

### 4.3. Ablation Experiments

The ablation experiments were conducted in order to validate the effectiveness of the proposed improvements. RT-DETR-r18 (depth = 0.33, width = 0.25) was chosen as the baseline model. The following modifications were implemented: the 18-layer ResNet backbone was replaced with the IMNv4 backbone; GSConv was employed to replace specific SCs within the encoding module; the DDST module was utilized to replace the RepC3 module; the MPDSIoU loss was applied instead of the GIoU loss. Through these operations, the effect of each improvement was validated, and the results are presented in Table 4.

While utilizing the IMNv4 backbone network independently results in a 0.5% decrease in $AP_{0.5:0.95}$, it markedly enhances the model’s lightweight functionality, accompanied by a notable decline in the number of parameters and computational expense. Furthermore, the GConv reduces the number of parameters without compromising accuracy, while the DDST module enhances accuracy without increasing the number of parameters. The combined use of the GConv and DDST modules within the network yields a more pronounced effect. The MPDSIoU loss function enhances accuracy, thereby compensating for the decline in accuracy that is inherent to the model’s lightweight nature. In comparison to the baseline model, the STar-DETR proposed in this paper exhibits a 1.5% enhancement in $AP_{0.5:0.95}$, a decrease in Params to 5.13M (a 64.8% reduction), a decrease in FLOPs to 19.8G (a 41.8% reduction), and an increase in FPS to 125.8. The model demonstrates significant enhancements in both accuracy and efficiency, substantiating the efficacy of the proposed improvements.

To further validate the impact of the improved model on hardware resource usage during the inference phase, we selected the official RT-DETR-r18, the baseline model, and STar-DETR for batch inference and single-image inference on the GPU. The experimental results are presented in Table 5.

The lightweight improvements in this study primarily target computational complexity and memory usage. As evidenced by the results, the lightweight enhancements in STar-DETR effectively reduce GPU resource utilization, shorten inference time, and improve detection efficiency. However, since the CPU is primarily responsible for tasks such as data loading and task scheduling, its utilization rate remains largely unchanged.

### 4.4. Experiments on Key Innovations

#### 4.4.1. DDST Module Heatmap Experiments

**Figure 9:**
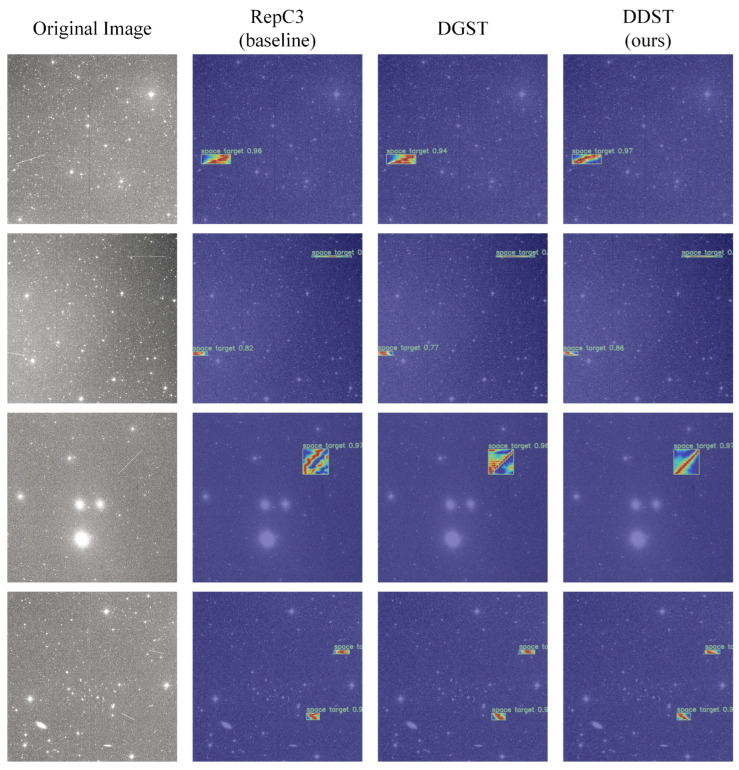
The results of heatmap experiments.

The RepC3 module in the baseline model was replaced with the DGST module and the improved DDST module for heatmap comparison experiments in order to demonstrate that the DDST module proposed in Section 3.3 can more effectively focus on the target areas. Heatmaps are employed for the visualization of the model’s attention to disparate regions of the image, with gradient colors denoting the model’s confidence in the presence of a target in a specific area. Figure 9 illustrates the detection result in heatmaps for the three networks.

In Figure 9, the red areas represent high confidence, indicating that the model considers these regions more likely to contain a target. While the blue areas represent low confidence, meaning the model believes these regions are less likely to contain a target. It can be observed that the overall detection box confidence is comparable across different modules; however, there is a discernible discrepancy in the model’s focus on distinct regions within the detection boxes. The application of the DDST module results in a greater concentration of red areas along the diagonal of the box, which corresponds to the space target trajectory. In contrast, when the DGST or RepC3 modules are employed, the red areas are more dispersed and cover a larger area. This illustrates that the DDST module is capable of learning the desired features and focusing on the space target trajectory, whereas other models may allocate more attention to regions that are farther from the features, resulting in model redundancy.

#### 4.4.2. IoU Experiments

In order to demonstrate the superiority of the MPDSIoU proposed in Section 3.4, several IoU loss functions that have been commonly employed in recent years were selected for comparison experiments. Except for the loss function, all other parts of the model remained the same, with the improved methodologies outlined in this paper being employed.

**Figure 10:**
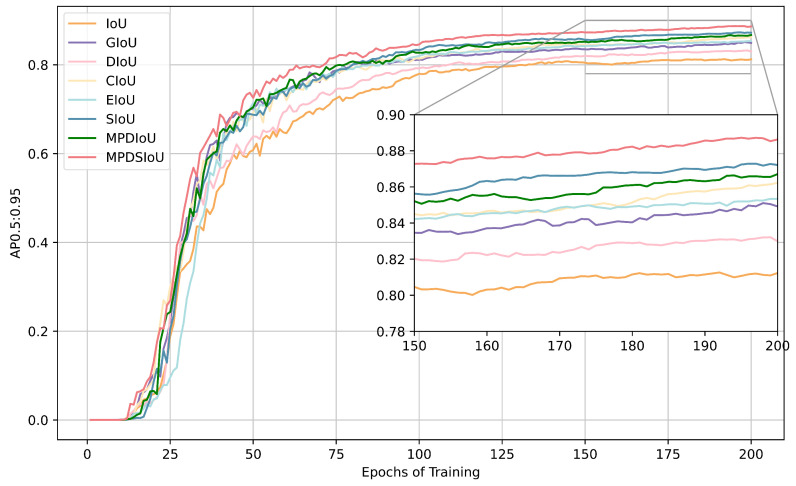
Accuracy convergence curves for different IoU loss functions.

Figure 10 illustrates the accuracy convergence curves of various IoU loss functions throughout the training process. To facilitate the interpretation of the results, a comparison of the training performance over the initial 200 epochs is presented. The experimental results demonstrate that MPDSIoU exhibits the most optimal performance in terms of both convergence speed and accuracy. With regard to accuracy, MPDSIoU is observed to outperform the second-placed SIoU by approximately 2% and the baseline GIoU model by approximately 4% during epochs 150–200. In terms of convergence speed, MPDSIoU displays a slight advantage. Conversely, traditional IoU and DIoU exhibit a notable delay in convergence speed by epoch 50, resulting in lower accuracy after the same number of epochs. The convergence speeds of the other loss functions exhibit only a slight discrepancy. The experimental results substantiate that MPDSIoU exhibits optimal performance.

### 4.5. Comparative Experiments

A comparative analysis was conducted of the proposed STar-DETR with 15 other object detection models on the test dataset. The general object detection models include faster R-CNN [51], cascade R-CNN [52,53], TOOD [54], ATSS [55], GFL [56,57], RetinaNet [58], RTMDet [59], YOLOX [60], YOLOv8, YOLOv10 [41], YOLOv11 [61], and RT-DETR [29]. The space target detection models proposed in the last two years include CS-YOLOv5 [26], AgeDETR [27], and enhanced YOLOv8 for space targets [4]. While spatial–temporal methods, such as SDebrisNet [18], have demonstrated effectiveness in video-based detection tasks, they are not directly applicable to our long-exposure single-frame dataset. Therefore, we have excluded such methods from our comparative analysis. The results of the experiments are presented in Table 6.

**Figure 11:**
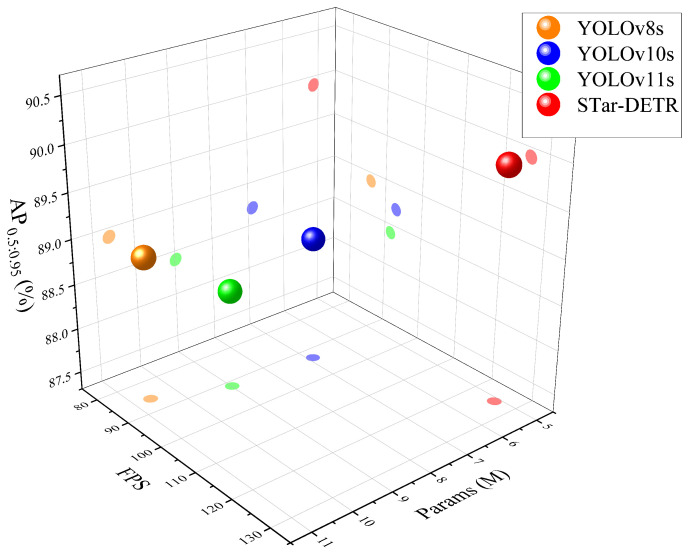
Three-dimensional scatter plot of model performance across multiple evaluation metrics.

The experimental results demonstrate that traditional two-stage detection models (e.g., faster R-CNN and cascade R-CNN) possess considerable parameter sizes and computational costs, which impede efficiency and render them unable to meet the demands of real-time detection. The improved models based on YOLO and DETR, such as CS-YOLOv5, AgeDETR, and enhanced YOLOv8 for space targets, have achieved accuracy improvements. However, further optimization is needed in terms of model computation and running efficiency. Among the latest YOLO models, YOLOv10s achieves the best balance between accuracy and efficiency. The proposed STar-DETR achieves an $AP_{0.5:0.95}$ of 89.9%, which represents a 1.2% improvement over YOLOv10s. With regard to Params and FLOPs, STar-DETR is 87.4% and 63.1% of YOLOv10s, respectively, while also exceeding it by 4.6% in FPS. To provide a more intuitive visualization of the differences in the model evaluation metrics, a three-dimensional scatter plot was constructed, comparing STar-DETR, YOLOv8, YOLOv10, and the recently proposed YOLOv11. As shown in Figure 11, the three axes represent Params, FPS, and $AP_{0.5:0.95}$. The models situated higher along the vertical axis indicate superior accuracy, whereas those positioned towards the right represent smaller parameter sizes and faster inference speeds. It is illustrated that the STar-DETR model offers notable advantages in terms of accuracy, efficiency, and real-time performance, rendering it well-suited for deployment on resource-constrained devices and enabling high-precision, rapid identification in practical applications.

**Figure 12:**
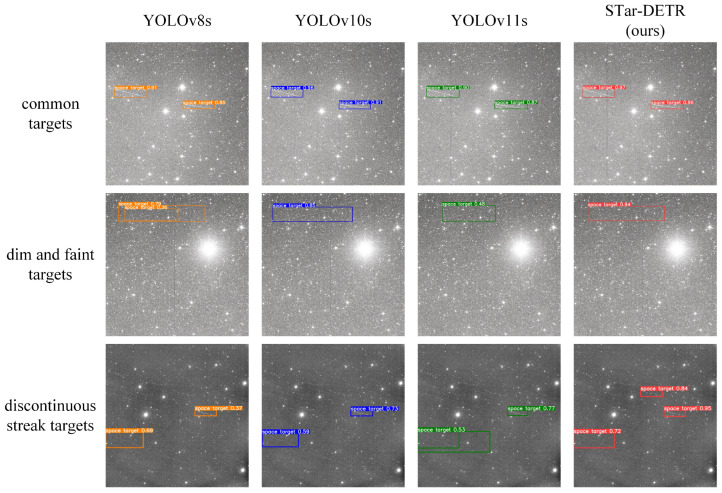
Detection results of YOLOv8s, YOLOv10s, YOLOv11s, and STar-DETR for different kinds of space targets.

A comparison of the detection results for YOLOv8s, YOLOv10s, YOLOv11s, and STar-DETR is shown in Figure 12. In the context of space target detection, the majority of observed targets are common space targets. However, some space targets, due to their considerable distance from the observer, diminutive size, and poor reflectivity, appear as dim and faint targets with less well-defined trajectories. Furthermore, some telescopic equipment employs intermittent exposure observation modes, resulting in discontinuous streak targets, whereby the trajectories appear as discontinuous lines on a straight path. Figure 12 presents the detection results for these three types of space targets across different models, with the detection box colors corresponding to those in Figure 11. With regard to common targets, all models display excellent detection performance, with STar-DETR exhibiting a slight advantage in terms of detection confidence. In the case of dim and faint targets, STar-DETR demonstrates superior detection capability, while other models present issues such as overlapping detection boxes, positional offsets, and low detection confidence. As for discontinuous streak targets, STar-DETR maintains strong detection performance, while other models fail to detect some targets.

## 5. Conclusions

This paper proposes the lightweight STar-DETR model for space target detection, based on RT-DETR, with the goal of achieving a lightweight structure while maintaining detection accuracy. First, the IMNv4 backbone network is developed to extract high-quality features while significantly reducing parameters and computational complexity. Second, the lightweight GSConv is integrated into the efficient hybrid encoder to reduce parameters while preserving the continuity of semantic information propagation in the SC. This improves the model’s ability to represent non-linear features and enhances information exchange between channels. Subsequently, the DDST feature fusion module is introduced, emphasizing the trajectory of space target exposure during multi-scale feature fusion, thereby mitigating overfitting issues. Finally, the MPDSIoU localization loss function is proposed to account for multiple factors, including angle, shape, center distance, and vertex distance between the detection box and the GT box. This method enhances regression accuracy and accelerates model convergence.

A space target dataset is constructed in this study, combining offline and online data augmentation techniques to enhance model performance and robustness under diverse sensing conditions. Experimental results show that the STar-DETR model outperforms other models in detection efficiency, achieving significant lightweight optimization while further enhancing accuracy. The model meets the detection requirements for both faint space targets and discontinuous streak targets. This achievement enables the potential for practical deployment of the model on optical observation equipment, laying a foundation for the engineering application of space target detection technology.

Despite its promising performance, our proposed STar-DETR has certain limitations. Specifically, it is designed for single-frame detection and does not incorporate spatial–temporal modeling, which may be advantageous for video-based detection tasks. Future work could explore the integration of spatial–temporal features to enhance the model’s capability in handling more complex scenarios, such as continuous image sequences or video data. Additionally, STar-DETR remains in the experimental phase and has not been applied to astronomical observation equipment. Therefore, in future work, we plan to conduct experiments on various hardware platforms and deploy the space target detection model in optical sensor systems. This will allow us to validate the model’s effectiveness, make further adjustments, and achieve an integrated system for image acquisition and space target detection.

## Figures and Tables

**Table 1:** The structure and parameters of the IMNv4 backbone.

Structure		Kernel		Channels			Stride	Paras	
**Layer**	**Block**	**DW** $K_{1}$	**DW** $K_{2}$	**Input**	**Mid**	**Output**		**Conv**	**BN.Weight** **& Bias**
Stem Layer	ConvBNAct	3 × 3		640 × 640 × 3	-	32	2	864	64
Layer 1	Fused IB	3 × 3		320 × 320 × 32	32	32	2	10,240	128
Layer 2	Fused IB	3 × 3		160 × 160 × 32	96	64	2	33,792	320
Layer 3	ExtraDW	5 × 5	5 × 5	80 × 80 × 64	192	96	2	37,120	1088
	IB	-	3 × 3	40 × 40 × 96	192	96	1	38,592	960
	IB	-	3 × 3	40 × 40 × 96	192	96	1	38,592	960
	IB	-	3 × 3	40 × 40 × 96	192	96	1	38,592	960
	IB	-	3 × 3	40 × 40 × 96	192	96	1	38,592	960
	ConvNext	3 × 3	-	40 × 40 × 96	384	96	1	74,592	1152
Layer 4	ExtraDW	3 × 3	3 × 3	40 × 40 × 96	576	128	2	135,072	2752
	ExtraDW	5 × 5	5 × 5	20 × 20 × 128	512	128	1	147,072	2560
	IB	-	5 × 5	20 × 20 × 128	512	128	1	143,872	2304
	IB	-	5 × 5	20 × 20 × 128	384	128	1	107,904	1792
	IB	-	3 × 3	20 × 20 × 128	512	128	1	135,680	2304
	IB	-	3 × 3	20 × 20 × 128	512	128	1	135,680	2304
	FFN	-	-	20 × 20 × 128	512	256	1	196,608	1536
Sum of Paras = 1,335,008 (1.273 M)									

**Table 2:** Experimental environment and parameter settings of RT-DETR.

Experimental Environment		Parameter Settings of RT-DETR	
Operating system	Windows Server 2022 Standard Evaluation	Batch size	32
CPU	Intel(R) Xeon(R) Silver 4210R CPU @ 2.40 GHz (Intel, Santa Clara, CA, USA)	Image size	640 × 640
GPU	NVIDIA Tesla T4 16G (NVIDIA, Santa Clara, CA, USA)	epochs	500
Development environment	Python 3.8.19 Pytorch 2.3.0 CUDA 11.8	Optimizer	AdamW
		Initial learning rate	0.0002
Compiler	VScode 1.87.2	Momentum	0.9

**Table 3:** The results of baseline model experiments.

Models	${\mathrm{AP}}_{0.5:0.95}$ (%)	Params (M)	Weights File (MB)	FLOPs (G)	FPS	Reference
RT-DETR-L	88.7	30.5	66.2	103.4	32.02	-
RT-DETR-r34	88.4	29.67	63	88.8	44.24	-
RT-DETR-r18	89.2	18.95	40.6	56.9	58.62	-
RT-DETR-r18 (depth = 0.67 width = 0.75)	89.1	16.72	35.6	45.2	71.01	YOLOv10m
RT-DETR-r18 (depth = 0.33 width = 0.5)	88.9	15.31	32.5	37.9	84.41	YOLOv10s
RT-DETR-r18 (depth = 0.33 width = 0.25)	88.4	14.58	30.9	34	90.9	YOLOv10n

**Table 4:** The results of ablation experiments.

IMNv4	GSConv	DDST	MPDSIoU	${\mathrm{AP}}_{0.5:0.95}$ (%)	Params (M)	Weights File (MB)	FLOPs (G)	FPS
-	-	-	-	88.4	14.58	30.9	34	90.9
✓	-	-	-	87.9	5.19	11.3	19.9	125.08
-	✓	-	-	88.4	14.51	30.8	33.9	90.07
-	-	✓	-	89.8	14.56	30.8	34	92.96
✓	✓	-	-	88	5.14	11.2	19.8	121.92
✓	-	✓	-	89.2	5.18	11.3	19.9	122.57
-	✓	✓	-	90	14.5	30.8	33.8	91.14
✓	✓	✓	-	89.4	5.13	11.2	19.8	125.8
✓	✓	✓	✓	89.9	5.13	11.2	19.8	125.8

**Table 5:** The results of hardware experiments.

Models	GPU Memory Usage (MiB)	CPU Utilization (%)	Inference Time (ms)	
			**Per Image**	**Single Image**
RT-DETR-r18	7019	20.2	17.1	44.2
RT-DETR-r18 (depth = 0.33 width = 0.25, baseline)	4031	20.1	11	29.7
STar-DETR (ours)	3156	20.1	7.9	24.7

**Table 6:** Performance comparison results of different models on the test dataset of space targets.

	Models	Backbone	Loss Function	${\mathrm{AP}}_{0.5:0.95}$ (%)	Params (M)	FLOPs (G)	FPS
General object detection models	Faster R-CNN_r50	ResNet50	CIoU	85.6	41.35	90.9	11.3
	Cascade R-CNN	ResNet50	SmoothL1	85.8	69.15	119	10.9
	TOOD	ResNet50	GIoU	80.9	32.02	78.8	11.1
	ATSS	ResNet50	GIoU	82.9	32.11	80.5	11.3
	GFL	ResNet50	GIoU	83.2	32.26	81.7	11.3
	RetinaNet	ResNet50	L1	74.6	36.33	81.6	11.4
	RTMDet_tiny	CSPNeXt	GIoU	88.4	4.87	8	49.9
	YOLOX_tiny	CSPDarknet	IoU	74.1	5.03	7.6	51.6
	YOLOv8s	CSPDarknet	GIoU	88.9	10.61	28.4	85.8
	YOLOv10s	CSPDarknet	GIoU	88.7	6.88	21.4	93
	YOLOv11s	CSPDarknet	GIoU	88.4	8.98	21.3	91.6
	RT-DETR-r18	ResNet18	GIoU	89.2	18.95	56.9	58.6
Space target detection models	CS-YOLOv5	CSPDarknet	GIoU	86.8	15.57	27.4	56.2
	AgeDETR	EF-ResNet18	GIoU	89.7	15.14	47.9	50.5
	Enhanced YOLOv8 for space targets	CSPDarknet	GIoU	89.2	7.2	23.4	66.2
	STar-DETR (Ours)	IMNv4	MPDSIoU	89.9	5.13	19.8	125.8

## Data Availability

The datasets presented in this article are not readily available due to privacy agreements with the collaborating observatory. If needed, please contact the corresponding author.
